# Complications and downsides of the robotic total knee arthroplasty: a systematic review

**DOI:** 10.1007/s00167-022-07031-1

**Published:** 2022-06-18

**Authors:** Christian Nogalo, Amit Meena, Elisabeth Abermann, Christian Fink

**Affiliations:** 1 Gelenkpunkt - Sports and Joint Surgery, FIFA Medical Centre of Excellence, Olympiastraße 39, 6020 Innsbruck, Austria; 2 Research Unit for Orthopaedic Sports Medicine and Injury Prevention (OSMI), Medical Informatics and Technology, UMIT - Private University for Health Sciences, Hall in Tirol, Austria

**Keywords:** Total knee arthroplasty, Total knee replacement, Robotic, Complications, Disadvantage, Downside

## Abstract

**Purpose:**

The purpose of this systematic review is to describe the complications and downsides of robotic systems in total knee arthroplasty (TKA).

**Methods:**

A comprehensive search according to the PRISMA guidelines was performed across PubMed, MEDLINE, Cochrane Central Register of Controlled Trials, Scopus, and Google Scholar from inception until December 2021. All articles of any study design directly reporting on complications and downsides of the robotic system in TKA were considered for inclusion. Risk of bias assessment was performed for all included studies using the Cochrane risk of bias and MINORS score.

**Results:**

A total of 21 studies were included, consisting of 4 randomized controlled trials, 7 prospective studies and 10 retrospective studies. Complications of the robotic system were pin-hole fracture, pin-related infection, iatrogenic soft tissue and bony injury, and excessive blood loss. While, downsides were longer operative duration, higher intraoperative cost, learning curve and aborting a robotic TKA due to different reasons. Iatrogenic injuries were more common in the active robotic system and abortion of the robotic TKA was reported only with active robotic TKA.

**Conclusion:**

Robotic TKA is associated with certain advantages and disadvantages. Therefore, surgeons need to be familiar with the system to use it effectively. Widespread adoption of the robotic system should always be evidence-based.

**Level of evidence:**

IV.

## Introduction

Robotics was first introduced into orthopedics surgery in the 1980s to improve the accuracy in implant positioning, prosthesis alignment and to reduce the rate of complications compared to conventional manual techniques [28]. Robotic total knee arthroplasty enhances the surgeon’s preoperative planning capabilities and real-time intra-operative dynamic referencing to allow for continuous assessments of range of motion and ligamentous tensioning. The real-time intra-operative kinematic assessment allows the comparison between the osteoarthritic knee and the new prosthetic knee [22]. However, with a lack of long-term evidence comparing clinical and functional results with conventional TKA and associated with increased costs and longer operative time, the trust in robotic TKA is restricted [14]. We are now in a pivotal time when data are emerging on robotic technologies, which makes it prudent to objectively examine whether their potential benefits are being realized.

In orthopedic surgery, every procedure is associated with some complications. Hence, it is fundamental to systematically examine the complications to improve the understanding and decrease these complications. It is also necessary to systematically assess the complications of this new technology before it can be widely used. In a recent systematic review [35], pin-hole fractures were reported in robotic TKA. But, to authors’ knowledge, no systematic review is available that comprehensively described all reported complications and downsides of robotic TKA. Therefore, we performed a systematic review of studies that reported complications and downsides of robotic TKA. Based on the results of the study, surgeons can perform appropriate risk stratification, which can help to counsel patients and their families, when selecting patients for robotic TKA. The hypothesis was that robotic TKA is associated with certain complications and downsides.

## Materials and methods

This systematic review followed Preferred Reporting Items for Systematic Reviews and Meta-analysis (PRISMA) Guidelines for literature search and it was registered on the PROSPERO International prospective register of systematic reviews (ID CRD42022303970).

### Search strategy

A literature review was performed using a strategy search designed to collect articles regarding complications and flaws of robotic TKA. Two independent authors (A.M., C.N.) conducted a comprehensive search across multiple databases (PubMed/MEDLINE, Cochrane Central Register of Controlled Trials, Scopus, and Google Scholar) and reviewed each article’s title and abstract for studies available until December 2021. Identified articles and their corresponding references were also reviewed according to selection criteria for additional eligible articles. The keywords used for initial screening were ((TKA) OR (TKR) OR (Total Knee Replacement) OR (Total Knee Arthroplasty)) AND ((Robot) OR (Robotic) OR (Robotic Surgical Procedure) OR (Robotic Arm Assisted)) AND ((Complication) OR (Disadvantage)). The full texts of the articles were obtained and evaluated when eligibility could not be assessed from the title and abstract. The eligibility of studies was independently assessed by these two authors and disagreements were resolved by consensus discussion between the authors, and a third author (C.F.) was consulted if the disagreement could not be resolved.

### Inclusion and exclusion criteria

All articles of any study design directly reporting on complications and downsides of the robotic system in TKA were considered for inclusion. Intraoperative complications, such as pin-hole fracture, iatrogenic soft tissue and bony injury, excessive blood loss, longer operative duration, aborting a robotic TKA due to different reasons and postoperative issues, such as pin-related infection, longer hospital stays and post-TKA stiffness, were noted. Any other complications and downsides of this technology were also reported. Non-English studies, non-peer-reviewed studies, review articles, case reports, surgical techniques, conference abstracts, non-clinical studies as well as studies solely focused on robotic unicompartmental knee arthroplasty, computer-assisted total knee arthroplasty, or navigated total knee arthroplasty were excluded.

The Cochrane risk of bias (RoB1) tool for randomized controlled trials [12] and methodological index for non-randomized studies (MINORS) tool [33] for observational studies was used for the quality assessment of the included studies. Two authors (A.M, C.N.) independently performed the quality assessment for each article. A third author (C.F.) was consulted in case of disagreement.

## Results

**Fig. 1:**
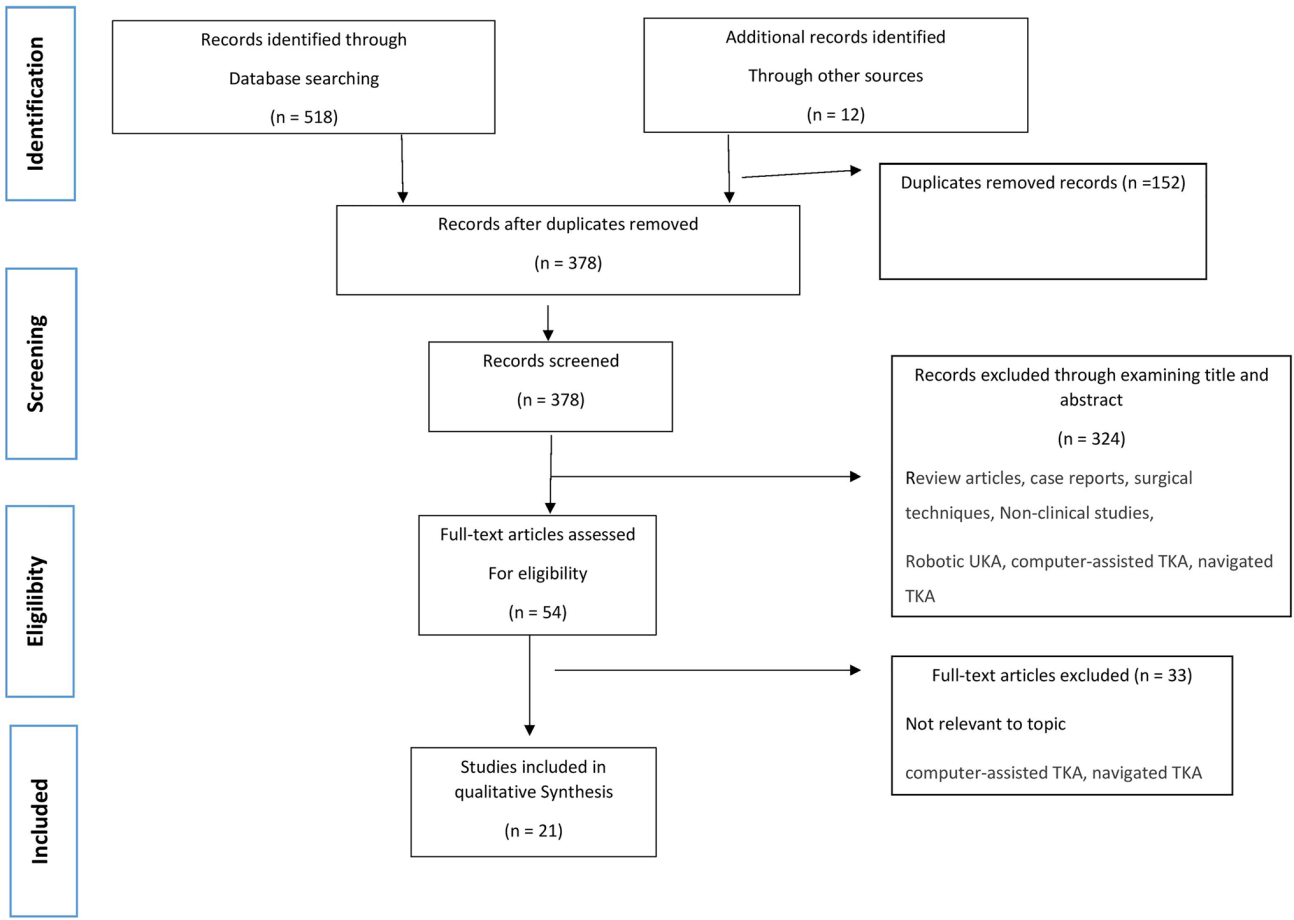
A selection process flow diagram to identify included studies

There were 530 potentially relevant studies identified in the initial comprehensive search across multi-databases and reference lists. Following the elimination of duplicate titles, abstracts were screened and pre-defined inclusion and exclusion criteria were applied. A large proportion of robotic-assisted surgeries was performed on unicompartmental knee arthroplasty (UKA) instead of TKA and hence, these studies were also excluded. A further 33 studies were excluded from analysis for various reasons after review of 54 full-text articles and one of the main reasons for exclusion was that data about complications were not available. Through discrete screenings, a total of 21 studies were included (Table 1), consisting of 4 randomized controlled trials, 7 prospective studies and 10 retrospective studies. A selection process flow diagram to identify included studies is presented in Fig. 1.

**Table 1:** Baseline characteristics included studies, (A) Semi-active system, (B) Active system, (C) Robotic system not mentioned

Table 1 Author			Year			Study design			Device/System			Level of evidence			Aim			Study subjects			Complication/ Downside		COI	
(A) Semi-active system																								
Bollars et al.			2019			Retrospective study			Smith & Nephew: NAVIO imageless surgical system, Memphis, TN, USA; semi-active System			IV			To determine whether there is a difference in the alignment accuracy between robotic and conventional TKA? Does the robot achieve the desired MA as planned by the surgeon’s preference?			77 RATKA and 77 MTKA			*Time: significant longer operation time in the RATKA group		YES	
Cotter et al.			2020			Retrospective study			Stryker comp.: MAKO Surgical Corporation, Kalamazoo, MI; semi-active System			IV			To compare 90-day episode-of-care (EOC) costs for MTKA and RATKA			147 RATKA and 139 MTKA			*Cost: Per minute operating cost was $14.44. RATKA group associated with longer surgical time. Higher intraoperative cost for RATKA vs. MTKA ($10,295.17 vs. 9,9998.78) Time: *Mean operative duration and mean total time spent in the operating room were significantly longer for RATKA group than MTKA (13.7 min vs 11.3 min)		YES	
Held et al.			2021			Retrospective study			Smith & Nephew: NAVIO imageless surgical system, Memphis, TN, USA; semi-active System			III			To investigate perioperative outcome, complications, and early patient-reported outcome measures (PROMs) of imageless RATKA system compared to conventional method			111 imageless RATKA and 110 MTKA			*Blood loss: RATKA cohort exhibited a statistically significant longer surgical duration (123 min vs. 107 min) and resultant greater estimated blood loss (240 mL vs. 190 mL). *Iatrogenic Injury: 1 RATKA patient had patellar tendon rupture that underwent surgical repair. *Pin Fracture: 1 minimally displaced, uni-cortical tibial shaft stress fracture at the pin site that was manage non-operatively *Pin Infection: 3 wound complications in RATKA group at the pin sites *Time: RATKA had a significant longer surgical duration compared to MTKA (123 min vs 107 min)		YES	
Marchand et al.			2020			Prospective cohort study			Stryker comp.: MAKO Surgical Corporation, Fort Lauderdale, FL, USA; semi-active System			III			To compare operative times for three cohorts during the first year following adoption of RATKA (initial, 6 months, and 1 year) and a prior cohort of manual TKA			60 RATKA and 60 MTKA			*Learning curve: appears to be the steepest in early stage of adaptation of the RATKA (1 month mean = 81 min, 6-month mean = 65 min)		YES	
Mitchell et al.			2021			Retrospective study			Stryker comp.: MAKO Surgical Corporation, Mahwah, NJ, USA; semi-active System			IV			To compare outcomes of MTKA and RATKA			148 RATKA and 139 MTKA			*Time: Mean tourniquet time was significantly longer in the RATKA group compared with MTKA (96.8 vs 91.6)		YES	
Naziri et al.			2019			Retrospective study			Stryker comp.: MAKO Surgical Corporation, Mahwah, NJ, USA, semi-active System			IV			To examining the learning curve associated with RATKA and comparing the surgeon’s robotics performance to his own traditional TKA			40 RATKA and 40 MTKA			*Time: RATKA required greater overall surgical time than MTKA (82.5 min vs. 78.3 min)		NO	
Savov et al.			2021			Prospective Case–control study			Smith & Nephew: NAVIO imageless surgical system, Memphis, TN, USA; semi-active System			III			To determine the learning curve necessary to minimize the time of surgery. To evaluate the accuracy of the implant alignment when using an imageless robotic system for TKA			70 RATKA and 70 MTKA			*Learning curve: for RATKA was completed after 11 cases		YES	
Smith et al.			2019			Prospective study			Stryker comp.: MAKO Surgical Corporation, Mahwah, NJ, USA; semi-active System			III			To determine if overall patient satisfaction can be improved with the use of robotic technology in TKA			120 RATKA and 103 MTKA			* Stiffness: 9 manipulations under anesthesia and 6 arthroscopic lysis of adhesions were performed in the RA-TKA group. *Time: Average total operative time was higher in RATKA group than MTKA (96 min vs 86 min)		NO	
Vermue et al.			2020			Retrospective study			Stryker comp.: MAKO Surgical Corporation, Fort Lauderdale, FL, USA; semi-active System			IV			To identify and predict the learning curve of RATKA			386 RATKA and 263 MTKA			*Learning curve: RA TKA was associated with a learning curve of 11–43 cases for operative time but not for the precision of limb alignment or component positioning. *Pin Fracture: 1 diaphyseal tibial stress fracture caused by the registration pin insertion. This healed uneventfully after 8 weeks. *Time: Operative time for the 10 RATKA cases were significantly longer than operative times prior to the introduction of the robotic system (RATKA 101.6 to 174.9 min vs 82.0 to 125.5 min)		NO	
Yun et al.			2021			Retrospective study			Stryker comp.: MAKO Surgical Corporation, Kalamazo, MI, USA; semi-active System			IV			To review the incidence of fracture with the conventional technique of bicortical diaphyseal pin placement. To evaluate a modified method of unicortical periarticular pin placement to mitigate this risk			1702 RATKA			*Pin Fracture: Femoral shaft fractures occurred in 3 patients. Fractures sites lied at the femoral array pin-hole. Fractures were treated with intramedullary femoral rodding		NO	
(B) Active system																								
Chun et al.																								
			2011			Retrospective study			Integrate Surgical systems Inc.: ROBODOC; Davis; CA, USA; active-System			IV			To elucidate and classify the causes of aborted RATKA and to find ways to reduce the incidence of these problems and to cope with them			62 RATKA			* Abortion: 22 RATKA abonded; 2 aborted during preoperative planning, 5 after patient anesthesia (before skin incision), 5 after surgical exposure (before milling) and 10 after milling began		N/A	
Jeon et al.			2019			Retrospective study			Curexo Technology Corporation, ROBODOC, Sacramento, CA, USA; active System			IV			To determine whether robot-assisted TKA improve clinical outcomes compared to the conventional procedure? Does robot-assisted TKA improve accurate alignment of components and does this accuracy lead to longevity of the implant over a long-term follow-up period?			94 RATKA and 334 MTKA			*Time: The mean tourniquet time was significantly different, being 45 min longer in the RATKA group than in the conventional TKA group (P < 0.001)		YES	
Kim et al.			2019			Prospective randomized, controlled study			Integrate Surgical systems Inc.: ROBODOC; Davis; CA, USA; active System			I			To compare RATKA to MTKA at long-term follow-up in terms of (1) functional results (2) radiographic parameters (3) Kaplan–Meier survivorship and (4) complications specific to RATKA			975 RATKA and 990 MTKA			*Blood loss: Intraoperative blood loss (mL) were higher in RATKA vs. MTKA (261 to 255). Intraoperative drainage volume was higher in RATKA vs. MTKA (798.1 vs 775) *Time: Longer operation and tourniquet time for RATKA vs. MTKA (97 min to 69 min; 75 min to 38 min)		NO	
Liow et al.			2016			Prospective randomized study			Integrate Surgical systems Inc.: ROBODOC; Davis; CA, USA; active System			II			To determine whether there was improvement in functional outcomes and quality-of-life (QoL) measures between RATKA and CTKA			31 RATKA and 29 MTKA			*Abandoned: RATKA has higher rate of complications, 7 complications observed in this study (5 RATKA vs. 2 MTKA) *RATKA aborted in 3 patients *Reason for abortion: one robot motor error, two technical error in the tibial work space		NO	
Mahure et al.			2021			Prospective cohort study			Tsolution One Total Knee Application; THINK Surgical System; active System			II			To assess the associated learning curve (LC) of active robot TKA			115 patients for active robotic TKA			*Learning curve: Operative time of active robotic TKA significantly decreases after 10 cases. Variable results with LC for each surgeon ranging from 12 to 20 cases. *One definitive device-related complication (metallic tack left within the distal femur) that occurred in a single patient *Stiffness: *3 RATKA patients had postoperative stiffness		NO	
Park et al.			2007			Prospective randomized study			Integrate Surgical systems Inc.: ROBODOC; Davis; CA, USA; active System			II			To compare RATKA and MTKA			32 RATKA and 30 MTKA			*Iatrogenic Injury: Complication in RATKA group: 1 patellar tendon rupture, 1 dislocation of the patella, 1 postoperative supracondylar fracture, 1 patellar fracture, and 1 peroneal injury *Pin Fracture: *1 postoperative supracondylar fracture in RATKA		N/A	
Siebert et al.			2002			Prospective study			U.R.S.-ortho GMbH & Co. KG, CASPAR, Rastatt, Germany; active System			III			To evaluate precision and accuracy of RATKA			70 RATKA and 52 MTKA			*Abortion: 1 RATKA aborted due to a defective registration marker *Learning curve: was noted with significantly longer operating times for the first cases. *Pin Infection: *3 RATKA patients had superficial skin irritations at the pin sites. *Time: longer operating time for RATKA for the first patients and almost normal operating times for later patients (135 v/s 90 min)		NO	
Song et al.			2012			Prospective randomized study			Integrate Surgical systems Inc.: ROBODOC; Davis; CA, USA; active System			I			To determine whether RATKA (1) improved clinical outcome; (2) improved mechanical axis alignment and implant inclination in the coronal and sagittal planes; (3) improved the gap balance; and (4) reduced complications			50 RATKA and 50 MTKA			*Iatrogenic Injury: In RATKA 2 patellar tendon abrasion. *Pin Infection: 1 RATKA patient had a seroma at the pin site. *Time: Mean operative time was 25 min longer for RATKA compared to MTKA group (99 min vs 74 min)		YES	
(C) Robotic system not mentioned																								
Antonios et al.			2019			Retrospective study			N/A (not available)			IV			To analyze trends in the use of technology-assisted TKA, identify factors associated with the use of these technologies, and describe potential drivers of cost			273,922 CATKA and 24,084 used RATKA			*Cost: Mean hospital charge for a RATKA has increased by 52.4% in the past 15 years		YES	
Kayani et al.			2018			Prospective cohort study			N/A			II			To determine learning curve for RATKA through assessments of operative times, surgical team comfort levels, accuracy of implant positioning, limb alignment, and postoperative complications. To compare accuracy of implant positioning and limb alignment in conventional jig-based TKA versus robotic arm-assisted TKA			60 RATKA and 60 MTKA			*Learning curve: RATKA was associated with a learning curve of seven cases for operative times and surgical team comfort levels *Pin Infection: 1 RATKA patient had minor wound dehiscence over the incision for the proximal tibial registration pins that was treated with regular dressings and prophylactic oral antibiotics		N/A	
Ofa et al.			2020			Retrospective cohort study			N/A			III			To investigate the differences between robotic TKA and non-robotic TKA on perioperative and postoperative complications and opioid consumption			5,228 RATKA and 750,122 MTKA			*Length of stay: Patients underwent RATKA had a longer hospital stay (4.38 vs 3.00)		NO

### Methodological quality assessment

The Cochrane risk of bias (RoB1) tool was used for the quality assessment of 4 RCT studies. The MINORS criteria were used for the quality assessment of 17 observational studies and the average score was 15.9, ranging from 8 to 20. The quality assessment data are shown in Tables 2 and 3.

**Table 2:**
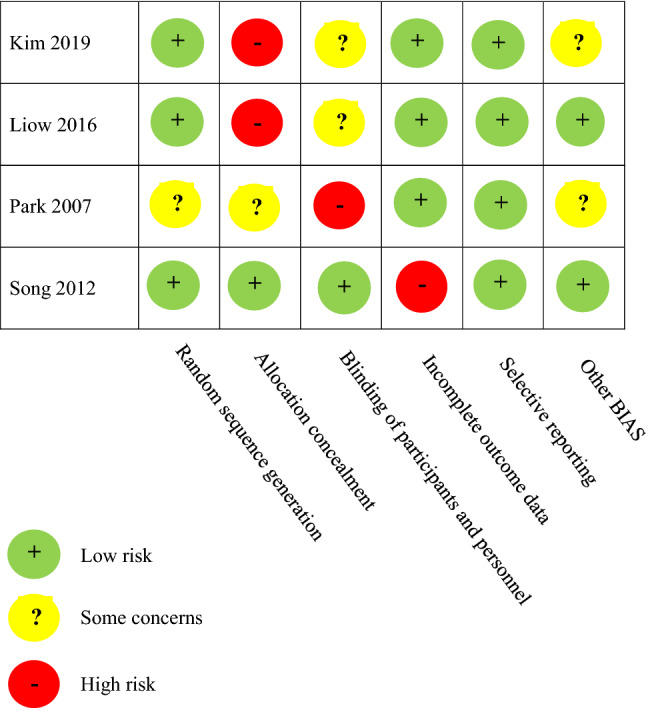
The Cochrane risk of bias (RoB) quality assessment data for RCT studies

**Table 3:** The MINORS criteria for the quality assessment of observational studies

Study	A clear stated aim	Inclusion of consecutive patients	Prospective collection of data	Endpoints appropriate to the aim of the study	Unbiased assessment of the study endpoint – BLIND	Follow-up period appropriate to the aim of the study	Loss to follow-up less than 5%	Prospective calculation of the study size –- CI	An adequate control group	Contemporary groups	Baseline equivalence of groups	Adequate statistical analyses	Total
Antonios et al.	2	2	2	2	1	0	0	2	0	2	2	2	17
Bollars et al.	1	2	2	2	2	1	0	2	0	2	2	2	18
Chun et al.	2	0	2	2	0	0	0	0	0	0	2	0	8
Cotter et al.	2	2	2	2	1	1	0	0	0	2	2	2	16
Held et al.	2	2	2	2	0	2	2	0	0	2	2	1	17
Jeon et al.	2	2	2	2	0	2	2	2	0	2	2	2	20
Kayani et al.	2	2	2	2	0	1	0	2	1	2	1	2	17
Mahure et al.	1	2	2	2	0	2	0	2	0	2	2	2	17
Marhand et al.	1	2	2	2	0	2	0	0	0	2	2	1	14
Mitchell et al.	1	2	2	2	2	2	1	0	0	2	2	2	18
Naziri et al.	2	2	2	2	1	2	0	0	0	2	2	2	17
Ofa et al.	1	2	2	2	1	1	0	1	0	2	2	2	16
Savov et al.	2	2	2	2	0	0	0	2	2	2	2	2	18
Siebert et al.	1	2	2	2	0	2	0	2	0	2	2	0	15
Smith et al.	1	2	2	2	2	2	0	0	0	2	2	2	17
Vermue et al.	2	2	2	2	0	0	0	0	0	2	2	1	13
Yun et al.	2	2	2	2	0	1	0	0	0	2	2	0	13

The items are score 0 (not reported), 1 (reported but inadequate) or 2 (reported and adequate). The global ideal score being 16 for non-comparative studies and 24 for comparative studies

Pin-hole fracture [11, 29, 38, 39], pin-related infection [11, 15, 32, 36], iatrogenic injuries [11, 29, 36], more blood loss [11, 17] in the robotic TKA and stiffness after robotic TKA [20, 34] were the reported complications. Whereas, longer operative duration [7, 9, 11, 13, 17, 24, 26, 32, 34, 36, 38], longer hospital stays [27], higher intraoperative cost [2, 9], learning curve [15, 20, 22, 31, 32, 38], aborting a robotic TKA due to different reasons [8, 19, 32] were reported downsides. Iatrogenic injuries were more common in the active robotic system and abortion of the robotic TKA was reported only with active robotic TKA. The results of included studies are illustrated in Table 4.

**Table 4:** Comparison of complications and downsides between MTKA and RATKA

Variable	Robotic system		MTKA	RATKA	MTKA	RATKA
Total patient			1,026,590 (96**.**7%)	33,724 (3.3%)		
Pin fracture						
Held et al.	Semi-active	Tibial shaft stress fracture	N/A	1 (0.9%)		
Park et al.	Active	Supracondylar fracture	N/A	1 (3.1%)		
Vermue et al.	Semi-active	Tibia stress fracture	N/A	1 (0.6%)		
Yun et al.	Semi-active	Femoral shaft fracture	N/A	3 (0.2%)		
Total pin fracture			N/A	6 (0.28%); *N* = 2100		
Pin infection						
Held et al.	Semi-active	Wound complications	N/A	3 (2.7%)		
Kayani et al.	Not mentioned	Wound complications	N/A	1 (1.6%)		
Siebert et al.	Active	Superficial skin irritations	N/A	3 (4.3%)		
Song et al.	Active	Seroma	N/A	1 (2.0%)		
Total infections			N/A	8; (2.7%); *N* = 291		
Iatrogenic injury						
Held et al.	Semi-active	Patellar tendon rupture	1 (0.9%)	1 (0.9%)		
Partk et al.	Active	Patellar tendon rupture	N/A	1 (3.1%)		
		Patella dislocation	N/A	1 (3.1%)		
		Post. supracondyl. Fracture	N/A	1 (3.1%)		
		Patella fracture	N/A	1 (3.1%)		
		Peroneal injury	N/A	1 (3.1%)		
Song et al.	Active	Patellar tendon abrasion	N/A	2(4.0%)		
Total injuries			1; *N* = 190	8 (4.1%); *N* = 193		
Stiffness						
Mahure et al.	Active	Postoperative	N/A	3 (2.60%)		
Smith et al.	Semi-active	Under anesthesia	9 (8.7%)	9 (7.50%)		
		Arthroscopic lysis	3 (2.9%)	6 (5.00%)		
Total patients			12 (*N* = 103)	18 (*N* = 235)		
Blood loss			mL	mL		
Held et al.	Semi-active		190	240		
Kim et al.	Active		255	261		
Average blood loss			222.5	250.5		
Time (minutes)			Theater time		Tourniquet time	
Bollars et al.	Semi-active		72.0	102.0	11.3	13.7
Cotter et al.	Semi-active		141.2	154.9	N/A	N/A
Held et al.	Semi-active		107.0	123.0	N/A	N/A
Jeon et al.	Active		N/A	N/A	N/A	45 min longer
Kim et al.	Active		69.0	97.0	38.0	75.0
Mitchell et al.	Semi-active		N/A	N/A	91.6	96.8
Neziri et al.	Semi-active		78.3	82.5	N/A	N/A
Siebert et al.	Active		N/A	90.0	N/A	45 min longer
Smith et al.	Semi-active		86.0	96.0	N/A	N/A
Song et al.	Active		74.0	99.0	N/A	N/A
Vermue et al.	Semi-active		82.0	101.6	N/A	N/A
Average time			88.7	105.5	47	61.8
Lenght of stay						
Ofa et al.	Not mentioned	Days	3.0	4.4		
Costs						
Cotter et al.	Semi-active		N/A	14.44$ extra per minute of operative time		
Antonios et al.	Not mentioned		N/A	Cost increased by 52.4% in last 15 years		
Learning curve						
Kayani et al.	Not mentioned	Completed after	N/A	7 cases		
Mahure et al.	Active	Completed after	N/A	10 cases		
Marchand et al.	Semi-active	Shorter OR Time	N/A	First 1 month = 81 min, After 6 months = 65 min		
Savov et al.	Semi-active	Completed after	N/A	11 cases		
Siebert et al.	Active	Shorter OR Time	N/A	Initial patients = 135 min, later on = 90 min		
Vermue et al.	Semi-active	Completed after	N/A	11–43 patients		
Abortion			*N* (%)	*N* (%)		
Chun et al.	Active	During pre-op planning	N/A	2 (2%)		
		After patient anesthesia	N/A	5 (5%)		
		After surgical exposure	N/A	5 (5%)		
		After milling	N/A	10 (10%)		
Siebert et al.	Active	During milling	N/A	1 (1.4%)		
Liow et al.	Active	Technical error	N/A	3 (12.5%)		
Total			N/A	26; *N* = 201		

## Discussion

The most important finding of this systematic review was that robotic TKA is associated with longer operative time. Iatrogenic injuries were more common in the active robotic system and abortion of the robotic TKA was reported only with active robotic TKA.

Both, robotic and conventional systems have their advantages and disadvantages. Advantages of robotic TKA include accurate placement of prosthesis which results in fewer outliers in the component positions, superior implant alignment accuracy, precise bony cuts, and soft tissue balancing which are all considered a prerequisite for good functional outcome and endurance in TKA. A recent meta-analysis found improved short-term patient-reported outcomes (KSS and WOMAC) in the robotic group compared to the conventional TKA group [41]. However, these advantages of better clinical scores, patient satisfaction, and implant survivorship remain to be confirmed in long-term follow-up [18].

Three different robotic systems are available, based on the amount of autonomy delivered to both the surgeon and the robot, which include passive, active and semi-active. In a passive robotic system, the surgeon has continuous and direct control, while, an active robotic system is completely independent of the surgeon for performing a designated task. Therefore, active robotic systems are associated with increased chances of iatrogenic soft tissue injury. To ensure accuracy and safety against iatrogenic soft tissue or neurovascular injury, semi-active systems developed which provide tactile feedback to the surgeon, thus, helping define specific boundaries (i.e., for surgical resection or safety). The major goals of a semi-active system are to prevent gross intraoperative errors and reduce deviations from the surgical plan to ensure a safe procedure with well-aligned components. Although constant efforts have been made to improve the robotic system and decrease the associated complication, certain complications and downsides have been reported in the literature.

Femoral or tibial shaft fracture due to mechanical weakness caused by the pinholes is one of the most dreaded complications of the robotic TKA. In their study of 385 TKAs Beldame et al. [4] found the incidence of pin-site femoral fracture fractures to be 1.4%. They found a unique pattern of pin-site fracture where fractures occur an average of 12.6 weeks after arthroplasty, and before fracture episodes, patients experienced unusual pain for several days in the thigh. These fractures were associated with minor or indirect trauma and all of them were treated by intramedullary fixation. Yun et al. [39] and Baek et al. [3] recommended periarticular pin placement because the bone at this site is more robust to torsional and bending stresses than the diaphysis. Vermue et al. [38] advised for the smaller pins to prevent this complication. Preoperatively, all the patients should be informed about the potential risk of pin-hole fractures because it is not rare. Pin-site infection is another specific complication of tracker pin that may require antibiotics and dressing for an additional duration. However, the incidence of pin-site infection was reported to be low in general (0.47%) [11].

Iatrogenic soft tissue and bony injuries include patellar tendon rupture, dislocation of the patella, patellar fracture, and peroneal nerve injury [29]. Patellar tendon rupture was also seen in the study by Held et al. [11] and the patient underwent surgical repair. Although Kayani et al. [16] reported that Robotic TKA was associated with reduced bone and soft tissue injury compared to conventional TKA, other studies documented the opposite [11, 29, 36]. Iatrogenic injures were more common in active robotic system. Therefore, surgeons should be aware and try to avoid any iatrogenic injury while taking bone cuts.

In a recent study, Held et al. [11] found greater estimated blood loss in the robotic group which may be attributed to the prolonged operative time. In another long-term study by Kim et al. [17], the robotic group was associated with more blood loss and postoperative drainage volume. Bohl et al. [6] reported that longer surgical duration in hip and knee arthroplasty may require transfusion. However, it is important to recognize that robotic TKA does not require opening of the femoral canal which should theoretically result in less blood loss. Therefore, studies are needed with a large sample size to examine the difference in blood loss between these two groups.

Surgical robots are suggested to decrease post-TKA stiffness incidence by the accurate placement of a prosthesis and precise alignment but, this systematic review found studies that reported stiffness following robotic TKA. However, stiffness after TKA is multifactorial, in their recent systematic review, Zaffagini et al. [40] found modifiable and non-modifiable causes for post-TKA stiffness. Robots may help to correct the modifiable causes, such as prosthesis malalignment and overstuffing of joint but, other factors may not be corrected using robotic surgery.

This systematic review also identified some downsides of robotic systems compared to conventional instrumentation. The most consistent finding among different studies was longer intraoperative time with robotic systems. This longer duration is due to insertion and removal of pins in the femur and tibia, registration of the knee joint with the robotic system, and intraoperative planning. Longer surgical time is associated with a higher risk of infection which may result in devastating outcomes of TKA [25]. Pugely et al. [30] reported that after 120 min of surgical time the risk of infection increases to 1.8 times in joint arthroplasty. Two recent systematic reviews reported that the incidence of deep prosthetic joint infection was higher in robotic group at 1.6–1.7% compared to 0.44–1.0% in conventional TKA [21, 28]. Moreover, prolonged surgical duration is in part responsible for the higher intraoperative costs of robotic TKA. Other factors responsible for higher intraoperative costs were higher anesthesia costs, operation theater supplies, robotic maintenance costs, robotic-specific disposables costs, software requirements, and additional diagnostic imaging. On the other hand, some authors believe that robotic systems may improve TKA survivorship which would result in a decreased cost for future revision. However, to date, there is a lack of conclusive data on the relationship between the use of robotic systems and the longevity of TKA implants [1, 2, 10, 37]. Due to the additional time and expense associated with robotic systems in their long-term study, Kim 2019 et al. [17] did not recommend widespread use of robotic TKA.

There is conflicting evidence with respect to the length of hospital stay after robotic TKA. Most of the included studies reported shorter hospital stays in the robotic group, except for one study [27]. This was a nationwide database study from 2010 to 2017 which reported on significantly longer hospital stays following robotic TKA. Therefore, further research is needed for clarification of this matter.

Aborting a robotic TKA was another downside identified in this systematic review. Although all these studies used active robotic system. Regardless of the robotic system, reported abortion rates for robotic arthroplasty are between 1 and 12% [5]. Therefore, the surgeon should be aware of potential problems with the robotic system used, to avoid them upfront or to cope with them.

Another challenge with new technology is the learning curve. TKA robotics is associated with a learning curve that affects the comfort level of the surgical team. It has been shown that during this initial learning phase, the robotic system was associated with heightened levels of anxiety among the surgical team. This is an important consideration because stress and mental strain are correlated with diminished surgical performance, poor decision-making, and reduced technical skills [23]. The learning curve probably depends on the surgeon's previous experience and the general level of competence in robotics in surgery [20]. Therefore, surgeons starting with robotics TKA should foresee enough time to cope with this learning curve during initial cases.

This study has some key limitations. First, the heterogeneous approaches adopted in evaluating complications and downside of the robotic system did not allow a meta-analysis of the retrieved data. Second, the selection criteria, such as the exclusion of robotic unicompartmental knee arthroplasty, computer-assisted total knee arthroplasty or navigated total knee arthroplasty may have excluded relevant studies. Third, there are few studies on robotic TKAs and most of them are with small sample sizes with short-term follow-up. Future studies with large sample sizes and long-term follow-up will be needed to provide more conclusive findings in assessing the complications and downside of this system. Analysis of the national registry data will be a key finding to look out for the relevant complication associated with robotic TKA. Fourth, these studies used different robotic systems in different populations so there may be bias in the reporting of the complication.

The ultimate goal of the TKA is to create a stable, painless, long-lasting joint, which may be achieved by both conventional and robotic-assisted methods. The surgeon should be aware that despite the potential advantages of the robotic system, this new technology, may be associated with certain complications and downsides. This emerging technology is a tool, available to surgeons and they should decide which techniques will provide them and their patients with the optimum outcomes.

## Conclusion

Robotic TKA is associated with certain advantages and disadvantages. Therefore, surgeons need to be familiar with the system to use it effectively. Widespread adoption of the robotic system should always be evidence-based.
